# Generalizing prosodic patterns by a non-vocal learning mammal

**DOI:** 10.1007/s10071-016-1036-8

**Published:** 2016-09-22

**Authors:** Juan M. Toro, Marisa Hoeschele

**Affiliations:** 1 ICREA, Pg. Lluis Companys 23, 08019 Barcelona, Spain; 2 Center for Brain and Cognition, Universitat Pompeu Fabra, Roc Boronat, 138, 08018 Barcelona, Spain; 3 Department of Cognitive Biology, University of Vienna, Althanstrasse 14, 1090 Vienna, Austria

**Keywords:** Prosody, Rats, Vocal learning, Operant conditioning

## Abstract

Prosody, a salient aspect of speech that includes rhythm and intonation, has been shown to help infants acquire some aspects of syntax. Recent studies have shown that birds of two vocal learning species are able to categorize human speech stimuli based on prosody. In the current study, we found that the non-vocal learning rat could also discriminate human speech stimuli based on prosody. Not only that, but rats were able to generalize to novel stimuli they had not been trained with, which suggests that they had not simply memorized the properties of individual stimuli, but learned a prosodic rule. When tested with stimuli with either one or three out of the four prosodic cues removed, the rats did poorly, suggesting that all cues were necessary for the rats to solve the task. This result is in contrast to results with humans and budgerigars, both of which had previously been studied using the same paradigm. Humans and budgerigars both learned the task and generalized to novel items, but were also able to solve the task with some of the cues removed. In conclusion, rats appear to have some of the perceptual abilities necessary to generalize prosodic patterns, in a similar though not identical way to the vocal learning species that have been studied.

## Introduction

From an early age, human infants take advantage of several sources of information to infer linguistic structure. For example, much research has demonstrated they can use statistical regularities to track dependencies among elements in speech (for a review see Aslin and Newport 2008). However, statistical regularities are only one of the cues infants might use to infer different aspects of language structure (e.g., Yang 2004). Another prominent source of information that is readily available in the signal is prosody. Prosody in speech refers to acoustic features that include pitch, intensity, duration, and timbre. Several lines of research suggest that prosody acts as an organizing parameter that is readily used by both infants and adults (e.g., Nespor and Vogel 2008) complementing other sources of information, such as statistical regularities. Infants as young as 6 months of age use changes in prosodic cues to identify noun and verb phrases (Soderstrom et al. 2003) and to learn new words (Shukla et al. 2011). Infants also readily use prosodic cues to infer word order in their language (Gervain and Werker 2013). Interestingly, infants do not need extensive experience with a given set of acoustic cues to take advantage of them. Both native and non-native prosody seem to help infants organize input, suggesting prosody’s role might be closely related to acoustic salience (Hawthorn et al. 2015; for similar results with adults see Langus et al. 2012; Shukla et al. 2007). Thus, humans use acoustic changes to organize the speech signal and to bootstrap linguistic structure (Nespor and Vogel 2008).

Research with non-human animals has shown that the ability to detect statistical and rhythmic information is not limited to humans. Baboons *Papio papio*; Minier et al. 2016), tamarins (*Saguinus oedipus*; Hauser et al. 2001), zebra finches *Taeniopygia guttata*; Lu and Vicario 2014) and rats (*Rattus norvegicus*; Toro et al. 2016) are able to track the same statistical dependencies that infants do among different elements in a sequence. The variety of species in which statistical learning abilities have been exhibited suggests that the mechanisms responsible for them are widely shared among vertebrates. Research also suggests that at least some species can effectively process rhythmic information. Parrots show evidence of detecting and synchronizing to a regular beat (Patel et al. 2009), and European starlings can be trained to discriminate among acoustic sequences based on rhythm (Hulse et al. 1984). However, rhesus monkeys do not seem to anticipate rhythmically organized events (Zarco et al. 2009), suggesting that the ability to correctly synchronize to rhythmic information may depend on vocal learning abilities (see Patel 2014; but see Cook et al. 2013, for evidence of rhythmic synchronization in a non-vocal learning species).

While rhythm is one aspect of prosody that seems to be detected by other species, only recently have studies explored prosodic processing using human speech in non-human animals. Recent studies with birds have unveiled their ability to detect prosodic changes and use them to differentiate between sequences. Zebra finches use changes in pitch, duration, and amplitude to discriminate between strings of syllables (Spierings and ten Cate 2014). Moreover, budgerigars can use differences in stress patterns to disambiguate words that were otherwise the same (Hoeschele and Fitch 2016). Budgerigars learned to discriminate words with an iambic stress pattern (where the last syllable is stressed) from words with a trochaic stress pattern (where the first syllable is stressed). Tests eliminating one or more cues from the signal (like pitch, duration, loudness, or vowel quality) showed that the budgerigars used a combination of them to make their discrimination. More importantly, tests with novel words demonstrated that they could generalize this discrimination to items they had not heard before. This suggests the birds’ performance was based on categorical perception of stress patterns and not merely on the memorization of the specific training sequences. For a direct comparison, the experiment was also conducted with humans with highly similar results (Hoeschele and Fitch 2016). Thus, two species of avian vocal learners (zebra finches and budgerigars) display a remarkable ability to detect and use prosody in speech as a differentiating cue much like humans do.

But, is the ability to use prosodic cues to discriminate between sequences widely shared across species distant in the phylogenetic tree, as it is for the ability to process statistical dependencies? If so, the prediction is that we should observe it in different taxa, independently of vocal learning abilities. On the contrary, if prosody processing is dependent on specialized traits, such as learning species-specific vocalizations, we should not observe it in mammals like rats, in which there is no evidence of vocal learning (e.g., Litvin et al. 2007). In the present study, we wanted to explore the extent to which a non-vocal learning species could identify and generalize across prosodic patterns. We presented rats with disyllabic consonant–vowel-consonant–vowel (CVCV) words where either the first or the second syllable was stressed. Using the same design and stimuli as the budgerigar and human study, we performed two tests: One assessing generalization to novel items and one assessing the cues used to process prosody. Using the same methodology allowed us to compare a non-vocal learner’s performance directly with that of two vocal learning species.

## Methods

### Subjects

Subjects were 24 female Long-Evans rats of 4 months of age. They were food-deprived until they reached 90 % of their free-feeding weight. They had access to water ad libitum. Food was administered after each training session. Half of the animals (*N* = 12) were assigned to the Iambic group and half (*N* = 12) to the Trochaic group.

### Stimuli

**Fig. 1:**
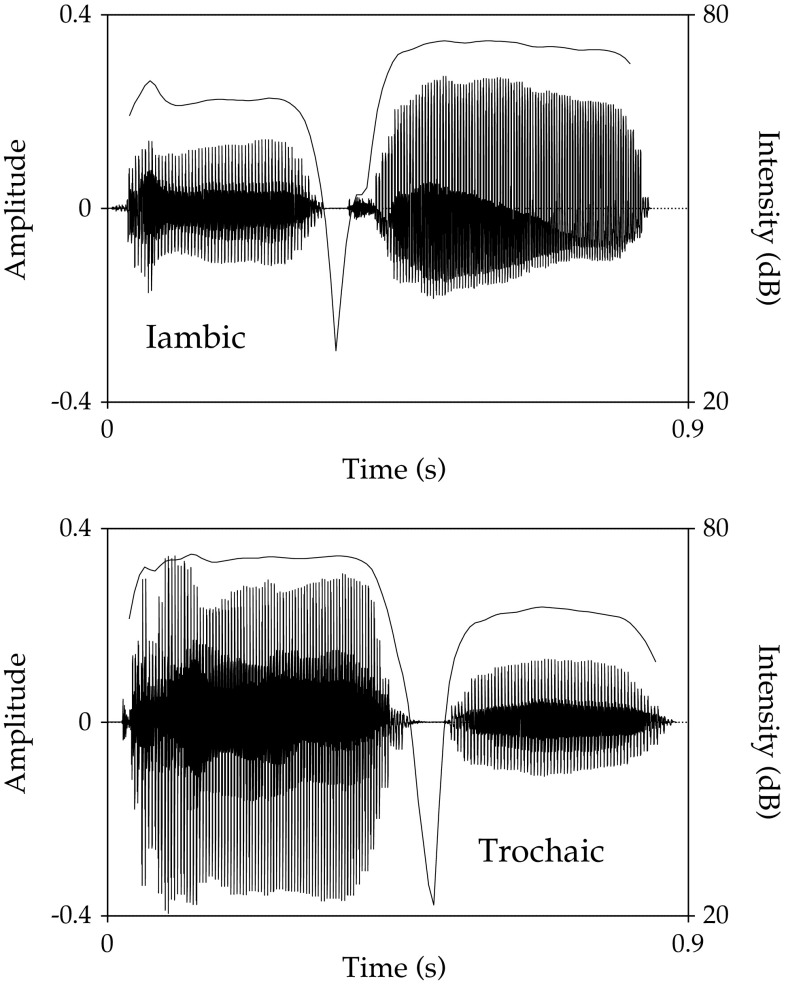
Waveforms illustrating the amplitude and intensity variations for the iambic and trochaic versions of the nonsense word/gapu/

We used the same stimuli as Hoeschele and Fitch (2016; all stimuli were previously included as open-access supplementary material which can be found here: http://link.springer.com/article/10.1007%2Fs10071-016-0968-3#SupplementaryMaterial) for both training and testing. Briefly, these were 24 CVCV nonsense words composed by combining 12 syllables (see below). There were two versions of each word, an iambic version (with stress on the second syllable) and a trochaic version (with stress on the first syllable; for an example, see Fig. 1). All stimuli were recordings of M.H. speaking in a flat tone, which were then artificially manipulated to produce stressed and unstressed syllables. To manipulate stress, we used four features: pitch, loudness, duration, and vowel quality. Only vowel quality was altered during the recording process (using common stressed and unstressed vowel pronunciations from English, see Hoeschele and Fitch 2016, for details on vowel types produced), and all other features were artificially manipulated. Unstressed syllables had an F0 of 194 Hz and a duration ranging randomly between 300 and 400 ms. Stressed syllables had an F0 ranging randomly between 230 and 280 Hz and a duration of 500 ms. Unstressed syllables were randomly between 7 and 10 dB quieter than stressed syllables and were produced with a short vowel sound, while stressed syllables were produced with a long vowel sound. Stimuli were presented at 68 dB (about 8 dB above rat threshold for the relevant frequencies; Heffner et al. 1994). All parameters of stimuli presentation were within those used in previous studies with rats and speech stimuli (e.g., Ahmed et al. 2011; Perez et al. 2013; Toro et al. 2016). There were a total of 48 different stimuli. We divided the words into two sets. Set 1 was composed of the iambic and trochaic versions of 12 words made from 6 of the 12 syllables (/ga/,/ke/,/na/,/pu/,/vo/,/zi/). All syllables were equally often used as the first and the last syllable, and no syllable occurred twice in the same word. Set 2 was composed by the iambic and trochaic versions of the other 12 words made from the other 6 syllables (/de/,/ji/,/lu/,/mi/,/su/,/to/). Half of the animals were trained with Set 1 and the other half with Set 2. Half of the animals were reinforced for responding to trochaic words and the other half for responding to iambic words.

To explore the specific cues the animals could be using for their discrimination, we created a set of test items in which some acoustic features distinguishing stressed and unstressed items were removed. These were the same words used during training, but we used eight different acoustic manipulations (pitch removed, duration removed, amplitude removed, and vowel quality removed, and also pitch only, duration only, amplitude only, vowel quality only stimuli). To remove features, we made stressed and unstressed syllables have the same values. To remove vowel quality, we used the stressed vowel quality for both the stressed and unstressed syllable. To remove pitch, we used the unstressed flat pitch contour (194 Hz) for both the stressed and unstressed syllable. To remove amplitude, we used the stressed amplitude (RMS of 0.1) for both the stressed and unstressed syllable. To remove duration, we used the stressed syllable length (500 ms) for both the stressed and unstressed syllable.

### Apparatus

Rats were individually placed in Letica L830-C Skinner boxes (Panlab S. L., Barcelona, Spain), which were each equipped with a lever and a pellet feeder. Acoustic stimuli were presented using Electro Voice (s-40) speakers located beside the boxes. A custom-made program (RatboxCBC) controlled the presentation of stimuli, recorded the lever-press responses, and provided reinforcement through the pellet feeder during the experiment.

### Procedure

#### Training

Animals were trained to press a lever to obtain food. Once rats had learned the target lever-pressing response, discrimination training began. During discrimination training, nonsense words were presented as acoustic stimuli. There was an inter-stimulus interval of 20 s. For the animals assigned to the Iambic group, pellets were delivered for responses following the presentation of any word with an iambic stress pattern. For the animals assigned to the Trochaic group, pellets were delivered for responses following the presentation of any word with a trochaic stress pattern. Reinforcement was set to a variable ratio schedule of five responses (±2), i.e., rats had to press the lever an average of five times in order to receive reinforcement. In each training session, each of the 24 words comprising either Set 1 or Set 2 was presented twice, for a total of 48 presentations. Stimulus presentation was balanced, so there were no immediate repetitions of the same stimulus. Also, no more than three reinforced or non-reinforced stimuli could occur in a row. Each training session lasted 28 min. When animals reached a discrimination ratio (DR; calculated by dividing the number of responses to reinforced stimuli by the total number of responses to all stimuli) of 0.8, we ran a generalization test.

#### Generalization test

For this test, we replaced 16 of the words presented during training with 16 words from the set the animals had not been exposed to (i.e., 16 words from Set 2 were used to test animals trained with Set 1 and vice versa). Eight of the test words had an iambic stress pattern, and eight had a trochaic stress pattern. These test words replaced eight iambic and eight trochaic words used during training. Presentation of the test words was balanced to avoid repetitions. No food was delivered after the presentation of test items.

#### Tests with cues removed

After the generalization test, animals received additional training to ensure all rats still were performing at a DR of 0.8 or higher. After this, we ran a new set of tests exploring the animals’ responses to stimuli with cues removed (pitch removed, duration removed, amplitude removed, vowel quality removed, pitch only, duration only, amplitude only, or vowel quality only). To collect enough data across all the different probe categories (eight in total), we ran three test sessions with these stimuli. As in the previous generalization test, in each session we replaced 16 of the training words with test items, with the same number of iambic and trochaic training words replaced. Stimulus presentation was balanced, so no more than two items from the same category followed each other. To assess animals’ performance during the test, we compared the mean number of lever-pressing responses to iambic test stimuli with the mean number of lever-pressing responses to trochaic test stimuli for each kind of test type. To analyze the results in each group (Iambic group, Trochaic group), we first ran an ANOVA with factors test type (vowel quality removed, pitch removed, amplitude removed, duration removed, vowel quality only, pitch only, amplitude only, duration only) and test item (iambic, trochaic). We then compared the responses to iambic test items and to trochaic test items within each test type.

## Results

### Training

All the animals learned to discriminate between the iambic and trochaic words used during training. Animals in the Iambic group reached the learning criterion within 42–64 sessions (*M* = 60.08, SD = 8.65). Animals in the Trochaic group reached the learning criterion within 48–73 sessions (*M* = 63.08, SD = 6.82).

### Generalization test

**Fig. 2:**
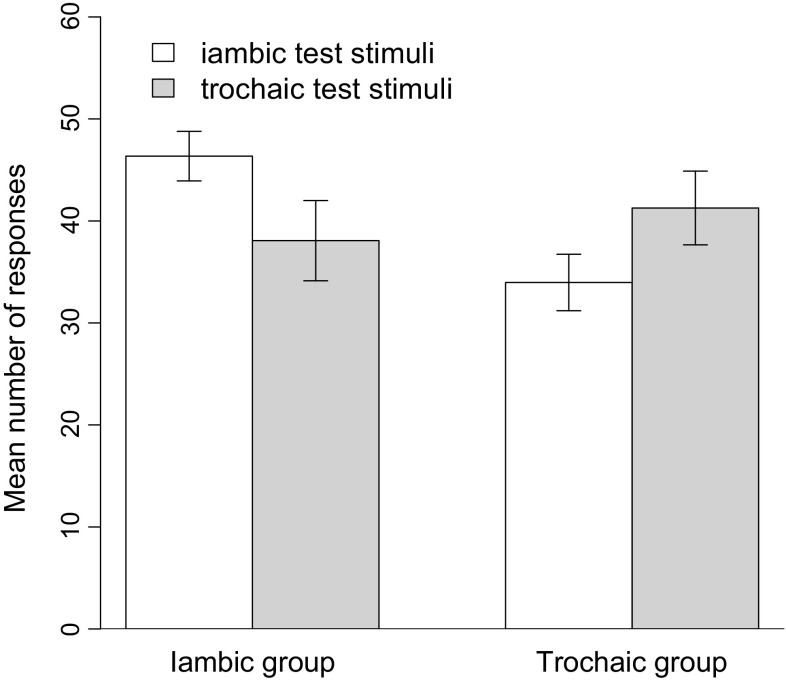
Mean number of responses to iambic (*white columns*) and trochaic (*gray columns*) test stimuli for the Iambic group and the Trochaic group during the generalization test

To assess whether the animals had memorized the training stimuli or had learned to distinguish iambic and trochaic words in general, we compared the mean number of responses in response to the novel words not presented during training. We have displayed these results in Fig. 2. Animals in the Iambic group responded significantly more [*t*(11) = 2.62, *p* < 0.05] to novel iambic test items (*M* = 46.35, SD = 8.42) than to novel trochaic test items (*M* = 38.06, SD = 13.61). Animals in the Trochaic group responded significantly more [*t*(11) = 3.38, *p* < 0.05] to novel trochaic test items (*M* = 41.26, SD = 12.51) than to novel iambic test items (*M* = 33.96, SD = 9.57). An analysis of variance with group (Iambic, Trochaic) and test item (iambic, trochaic) as factors revealed no main effect of group [*F*(1, 44) = 2.01, *p* = 0.163], or test item [*F*(1, 44) = 0.02, *p* = 0.88]. Importantly, there was a significant interaction between group and test item [*F*(1, 44) = 5.77, *p* < 0.05], confirming that animals in the Iambic group responded more to iambic test items than to trochaic test items, while animals in the Trochaic group responded more to trochaic test items than to iambic test items. Thus, the animals successfully generalized to new stimuli by differentially responding more to novel words that followed the learned stress pattern in both the iambic and the trochaic conditions.

### Tests with cues removed

In the second test, we wanted to explore the acoustic cues the animals were using to discriminate across stress patterns. For the Iambic group, an ANOVA with factors test (vowel quality removed, pitch removed, amplitude removed, duration removed, vowel quality only, pitch only, amplitude only, duration only) and test item (iambic, trochaic) revealed no main effects for test [*F*(7, 176) = 0.914, *p* = 0.497] or test item [*F*(1, 176) = 0.011, *p* = 0.916], and no interaction between them [*F*(7, 176) = 1.352, *p* = 0.229]. In fact, the mean number of responses to novel iambic or trochaic test items did not differ across test types (vowel quality removed: Iambic (*M* = 35.27, SD = 11.07), Trochaic (*M* = 32.5, SD = 7.96), *t*(11) = 1.68, *p* = 0.08; pitch removed: Iambic (*M* = 31.91, SD = 12.24), Trochaic (*M* = 38.52, SD = 14,62) *t*(11) = −1.52, *p* = 0.15; amplitude removed: Iambic (*M* = 40.52, SD = 13.7), Trochaic (*M* = 37.05, SD = 16.46) *t*(11) = 0.60, *p* = 0.55; duration removed: Iambic (*M* = 35.16, SD = 11.46), Trochaic (*M* = 32.8, SD = 19.77), *t*(11) = 0.37, *p* = 0.71; vowel quality only: Iambic (*M* = 30.8, SD = 7.64), Trochaic (*M* = 32.61, SD = 6.94), *t*(11) = −1.35, *p* = 0.201; pitch only: Iambic (*M* = 35.66, SD = 11.07), Trochaic (*M* = 32.5, SD = 7.96), *t*(11) = 1.01, *p* = 0.33; amplitude only: Iambic (*M* = 33.11, SD = 12.09), Trochaic (*M* = 41.16, SD = 17.78), *t*(11) = −1.94, *p* = 0.081; duration only: Iambic (*M* = 34.33, SD = 15.54), Trochaic (*M* = 28.58, SD = 14.14), *t*(11) = 1.91, *p* = 0.08). Animals assigned to the Iambic group could not discriminate test items that had one or several acoustic features removed.

Similarly, for the Trochaic group, no main effects were observed for test [*F*(7, 176) = 1.74, *p* = 0.102] or test item [*F*(1, 176) = 0.016, *p* = 0.9], and there were no significant interactions between them [*F*(7, 176) = 0.52, *p* = 0.813]. The mean number of responses to novel iambic or trochaic test items did not differ across test types (vowel quality removed: Iambic (*M* = 45.85, SD = 12.67), Trochaic (*M* = 38.3, SD = 9.69), *t*(11) = 1.617, *p* = 0.134; pitch removed: Iambic (*M* = 36.63, SD = 18.3), Trochaic (*M* = 43.3, SD = 15.38), *t*(11) = −1.75, *p* = 0.107; amplitude removed: Iambic (*M* = 44.11, SD = 14.06), Trochaic (*M* = 39.66, SD = 14.46), *t*(11) = 0.714, *p* = 0.489; duration removed: Iambic (*M* = 34.3, SD = 16.39), Trochaic (*M* = 38.94, SD = 18.96), *t*(11) = −0.877, *p* = 0.399; vowel quality only: Iambic (*M* = 39.83, SD = 18.62), Trochaic (*M* = 39.72, SD = 11.03), *t*(11) = 0.021, *p* = 0.983; pitch only: Iambic (*M* = 46.52, SD = 15.47), Trochaic (*M* = 46.27, SD = 11.26), *t*(11) = 0.051, *p* = 0.96; amplitude only: Iambic (*M* = 32.23, SD = 13.06), Trochaic (*M* = 33.49, SD = 23.92), *t*(11) = −0.223, *p* = 0.82; duration only: Iambic (*M* = 35.38, SD = 15.06), Trochaic (*M* = 37.44, SD = 13.4), *t*(11) = −0.50, *p* = 0.624). In contrast with the results observed in the generalization test, where we observed that the animals correctly discriminated novel stimuli that had the same prosodic pattern as the training words, here we show that the animals found it very difficult to discriminate stress patterns once acoustic cues were removed. It appears that the rats learned something about the prosodic contour differentiating reinforced from non-reinforced words during training because they were able to generalize it to new items. However, the rats appeared to require all prosodic cues to be presented together to discrimination iambic from trochaic stress.

## Discussion

Recent studies have shown two vocal learning bird species can use prosody to discriminate among sequences of speech (Hoeschele and Fitch 2016; Spierings and ten Cate 2014). In the present study, we wanted to explore whether such an ability would also be present in a mammal in which there is no evidence of vocal learning. Results show a remarkable use of prosodic stress by rats. All rats learned the task, and they were able to discriminate novel words based on prosodic pattern. However, and in contrast to what has been observed in budgerigars and humans in the same task (Hoeschele and Fitch 2016), rats failed to discriminate among test items when we removed individual acoustic cues including pitch, duration, intensity, and vowel quality.

Much like the rats in the current study, when budgerigars were presented with the same stimuli used here where all prosodic cues but one were removed (i.e., amplitude, duration, pitch, or vowel quality), they failed to discriminate between trochaic and iambic words (Hoeschele and Fitch 2016). Budgerigars could, however, discriminate between words in which only duration or pitch was removed (unlike the rats). Humans had little trouble discriminating trochaic and iambic words, unless they only contained vowel quality or duration information. Thus, it appears that the rats were the most impaired and the humans the least impaired when presented with stimuli with cues removed. The results we observe in the present study, that rats could not generalize their discrimination to test stimuli in which acoustic features were removed, shows they are highly sensitive to acoustic variations. Once we modified a salient acoustic feature in the present set of stimuli such as pitch, duration, intensity or vowel quality, discrimination fell to chance levels. This suggests that the representation of the stress pattern (iambic or trochaic) that the animals created during training and that allowed them to discriminate between novel test items was easily overridden by acoustic modifications (deleting either 1 or 3 features including pitch, duration, intensity, or vowel quality) that do not seem to be relevant for humans to solve this task. It is unlikely that the rats were focusing on a single aspect of the sound that was affected by all four manipulated features, because, for example, the interaction between pitch and vowel quality on the overall harmonic spectrum is different depending on the vowel type. Thus, it seems more likely the representation of stress that the rats formed required all features (pitch, duration, intensity, and vowel quality) to be present.

There are relatively few studies documenting the specific cues in human speech that non-human mammals might use for making discriminations; however, they often parallel human results. Ohms and collaborators (Ohms et al. 2012) showed that zebra finches assigned more importance to higher than to lower formants in a vowel discrimination task, paralleling results found in humans. Also similar to humans, trained chimpanzees are able to discriminate between words even when the acoustic signal is highly degraded, as when tested with noise-vocoded and sine-wave speech stimuli (Heimbauer et al. 2011). It has been found that rats, like humans, could use rise time to discriminate stimuli in a fricative-affricative continuum (Reed et al. 2003). Similarly, rats can learn to categorize vowels using temporal and spectral features just like humans (Eriksson and Villa 2006). These parallels suggest that at least some low-level cues present in speech are readily processed by non-human animals. Also, as we discussed in the introduction of the present study, there is evidence of non-linguistic rhythm processing across several species. The fact that rhythm synchronization has been observed in animals such as parrots (Patel et al. 2009) but not in monkeys (Zarco et al. 2009) suggests that this may depend on vocal learning abilities (see Patel 2014; but see Cook et al. 2013). In the present study, we observed that rats could generalize the rhythmic pattern to novel items presented in the first test. However, once removing acoustic cues changed this rhythm information, discrimination failed. This suggests such information may be relevant even for animals for whom there is no evidence of vocal learning abilities.

The ability to detect some prosodic cues in speech has been observed in other mammals before. We know that human newborns, cotton-top tamarin monkeys, and rats can use linguistic rhythm to discriminate among languages differing in rhythmic class (e.g., Dutch, a stress-timed language, from Japanese, a mora-timed language; Ramus et al. 2000; Toro et al. 2003). This is a remarkable finding, as it has been hypothesized that young infants might use linguistic rhythm to segment speech and start setting some syntactic parameters such as word order (e.g., Ramus et al. 1999; see also Nespor and Vogel 2008). If this hypothesis is true, then non-human animals have at least some of the prerequisite abilities humans use to analyze spoken language when they first encounter it. Similarly, rats can use differences in pitch and duration to group sequences of alternating tones following a pattern similar to that observed in human adults and infants described by the Iambic–Trochaic Law (de la Mora et al. 2013). Thus, the results we observe in the present study, together with previous studies of non-human animals, suggest that an ability fundamental to the acquisition of language in humans such as the one involved in prosodic processing is also present in other species. In addition, rats appear to have some of the perceptual abilities necessary to generalize prosodic patterns, much like the vocal learning species that have been studied so far (budgerigars and zebra finches). This suggests that extensive experience producing, processing, and learning complex vocalizations is not a necessary prerequisite to detect and use prosodic patterns as to discriminate novel stimuli.
